# Malaria in New England

**Published:** 1881-02

**Authors:** R. W. Griswold


### MALARIA IN NEW ENGLAND.

We clip the following rather novel theory in regard to malaria from the *Philadelphia Chemical News* of January 8, 1881:

There is going the rounds in the medical journals an extract from a paper in the *Michigan Medical News* on the present prevalence of ague and fever in portions of New England formerly and for a long time 'exempt from indigenous cases of socalled malarial fevers of any type.' Portions of the extract are as follows:

"It has been the boast of the New England states that they are free from fever and ague. * * * It is true malaria has prevailed in the history of these states, but the prevalence has been at long intervals. * * For forty years prior to 1865 fever and ague was unknown in that part of the Union. An epidemic of it followed the war of 1812, as also the war of the revolution; but between 1812 and 1865 there occurred no cases *de novo* in the New England states. These facts lead to the supposition that the germs of the malaria were imported by the soldiers returning to their homes from campaigns in malarial districts. * * During the past summer the disease has prevailed to an alarming extent, particularly along the Connecticut valley. In the Housatonic valley, in southwestern Massachusetts, * * * the malarial epidemic has been the severest ever known in New England * * The State (Mass.) Board of Health has undertaken an investigation into the causes of the unusual occurrence. * * * In the meantime conjecture is ripe * * * that the cause lies rather in the tainting of wells and water courses by the discharges of fever and ague patients than in the emanations from swamps or any other purely aerial or malarial agency."

My object in quoting this is to correct some errors in it as regards facts in reference to the existence of intermittent fever in the Eastern states in part, and in part to make some observations touching the etiology of the disease as it appears in this section. The statemement that "for forty years previous to 1865 fever and ague was unknown in that part of the Union" is not true, though as a general statement it has a basis of truth in it. If the writer of the paper in the *Michigan Medical News* excludes from his geography of New England the southwestern part of Connecticut, and speaks more of his own immediate locality (probably some part of Massachusetts), he may claim that his statement is in the main correct; but, geographically, politically and malarially, southwestern and middle Connecticut does not allow itself to be counted out of New England. The general statement that malarial diseases were nearly unknown for many years in New England is true. But explorers of old histories of diseases in Massachusetts and Connecticut find many records of the prevalence of ague and fever in different portions at different times. Dr. O. W. Holmes, on Intermittent Fever (1838), quotes Josselyn (1671) concerning the diseases of New England, in which "fever and ague" is twice mentioned. Hubbard's History of New England (1678) speaking of the settlement of New Haven, mentions fever and ague as among its diseases, and describes it in such way as to leave no boubt that it was intermittent fever, sometimes of a very severe type. The same history also speaks of the disease in towns west of New Haven. Cotton Mather, in his "Magnolia," tells of fever and ague, before his day, at Watertown and Cambridge, Mass. John Davenport, first minister of New Haven, speaks of ague and fever in his time at New Haven, Norwalk and Fairfield, Conn. The valleys of the Connecticut and Housatonic rivers showed malarious diseases soon after their first settlement by the whites. There were occasional outbreaks at different points during the 18th century. Coming into the first half of the 19th century, Dr. Holmes speaks of five towns in different sections of Connecticut, sixteen in Massachusetts, two in Rhode Island, one in New Hampshire and two in Maine where intermittent was thought to have originated previous to 1836. Dr. Wm. Buell, of Litchfield, Conn., in 1835, speaks of intermittent at Pittsfield, Mass., and New Milford, Conn., at an earlier date. Dr. Chief Justice Butler speaks of an epidemic of intermittent in and about Norwalk, Conn., from 1828 to 1832.

More than half of the population of Norwalk suffered from it at this time. In New Haven there were occasional cases between 1800 and 1810; in Stonington and North Stonington in 1837-8; and in Mystic and Old Lynn in 1863; from 1850 to 1856 there were many cases in New Haven and vicinity; and in 1853 in Norwalk, Fairfield and Bridgeport.

These relations, which I have made as brief as possible and have condensed from admirable papers on the subject by Dr. Henry Branson, of New Haven, President of the Connecticut Medical Society. in 1872, and Dr. A. W. Barrows, of Hartford, President of the Society in 1877, and which may be found in the Connecticut Medical Proceedings of those years, I have given here for the purpose in part of showing that "for forty years previous to 1865" fever and ague was not entirely unknown in New England, and that there were outbreaks of it in different sections and at different times not only after the wars of the revolution and of 1812, but at other periods as well; and that the statement that "between 1812 and 1865 there occurred no cases *de novo* in the New England states," is an error made by the writer in the *Michigan Medcial News* because of his ignorance of the medical history of the section. In part, also, I give these facts for the purpose of disposing of the absurd theory "that the germs of the malaria were imported by the soldiers returning to their homes from compaigns in malarial districts." I have heard that theory put forth many times before; a consideration of facts show that there is nothing in it. In New Haven, from 1850 to 1856; in Bridgeport, etc., in 1853, there were no soldiers to come home from campaigns and no campaigns in malarial regions to come home from. Further than this, although after the return of our soldiers from the army in 1865 there were cases of intermittent in the southwestern parts of this state, in the towns of the northern and eastern parts there were none, though they received their proportion of soldiers from the same regiments and the same regions of southern country. If returning soldiers "imported" intermittent into one corner of the state, why did they not also import it into the other corners? The theory is absurd.

Another point—the extent to which malarial diseases have prevailed in the valley of the Connecticut the past summer has been no more "alarming" than for the seven or eight years previous. Invading the lower part of the valley in 1871–72, intermittent has every year made a slow advance upward, and it is not until the last two years that it has *crawled* to the Massaohusetts line to any great extent. The like is true of the valley of the Housatonic; and a like fact is noticeable of the advance of the disease from the valley of the Connecticut, in the centre of the state, to the towns east of that river. Even up to the present time, malarial diseases are not recognized in the eastern towns of the state, thirty to eighty miles beyond where they have been extremely prevalent for seven years past. Observers note an advance northward and eastward of an average of about ten miles per year.

The statement that the malarial epidemic in south-western Massachusetts the past season "has been the severest ever known in New England," is no nearer the truth than some other statements noted. In the summer of 1876 of 368 persons living in a territory one mile long and one quarter of a mile or thereabout wide, in East Hartford, on the bank of the Connecticut river, opposite to the city of Hartford, 261 had ague and fever—figures given on the authority of Dr. M. Storrs, of Hartford. Other localities in the lower valley of the river can make showings not much if any below this; south-western Massachusetts will find it difficult to present a superior exhibit.

This brings into the consideration of the last half of my intention in this paper,—namely, some observations on the etiology of the disease. And first, as to the "tainting of wells and water courses by the discharges of fever and ague patients." In relation to water courses, we may set it down as a matter incontrovertible, that the source of a river cannot be tainted from its mouth; the adit is not poisoned from the exit: the fountain is not contaminated by emanations from the embouchure. Filth does not flow up stream from Long Island Sound into the hilly regions of Berkshire county per the bed of the Housatonic river, nor per the bed of the Connecticut, into Hampden. But it is the history of the present epidemic of ague and fever in those sections, that it has *gone up* the valleys of the streams, not only of the rivers mentioned, but also of the Naugatuck, from Bridgeport, and of the Quinnipiac, from New Haven. Upon what principal of hydraulics the contamination in these cases has traveled *in* the waters of the streams, philosophers will be puzzled to determine. In regard to the tainting of wells, the absurdity of the theory may not be so easily made apparent, since it is barely possible that contamination might be imparted to one well after another in a direct line through successive years, and over a long extent of ground, so as to produce the results that have been observed; but why, as in the valley of the Connecticut, contamination should go 90 miles north in a less time than 10 miles east could not be explained. And as there is no evidence as a basis of this theory of tainting wells, we may consider the doctrine of probabilities as presenting ten thousand chances against it to one in its favor. The "conjecture" mentioned in the *News* has no foundation on which to stand.

Here we are brought to the old theory of "malarial emanations." And what is malaria? Malaria is a suppositious factor believed to produce the phenomenon witnessed in intermittent and remittent fevers and other trouble. I have said *suppositious*, for the actual thing has not been grasped or determined, no one has ever isolated it or exposed it to view. Certain effects are observed, but the cause of those effects are an unknown quantity. The Board of Health of Massachusetts, which proposes to look after the causes of the appearance of ague and fever in the south-western corner of that state, will, unless it is different from any other health board ever heard of, discover, or will believe it discovers, the potentiality of that disease. It must report, and after it has reported we shall know all that we now know about it, and nothing more. The members of a board of health are not going to nose around the country in search of a cause, and report that their labors have not been without success. In one case they will find a neglected sewer, in another an untrapped drain, in a third a new pond, in a fourth an old pond dried up, in a fifth a damp cellar, in a sixth a pig pen saturating the soil in the direction of the well, in the seventh a cesspool, in an eighth a load of rotten garbage, in a ninth a bed of decayed leaves, and in a tenth a defecation—human, equine, bovine, canine, feline,—and in every case the *find* will be charged with the guilt of cause. I say the board will do this, predicated upon the fact that it is what all boards do. This will involve the deduction that all the things named, and a hundred other things, not named, are each and all capable of generating and evolving the poison that produces intermittent fever; and at the same time we shall be told that the poison of intermittent is a peculiar and *specific* poison, capacitated to produce only the especial class of diseases called malaria; also at the same time we shall be told by some other Board of inquirers that from each and every one of these things are begotten the *specific* poison of typhus fever, typhoid, dysentery, diarrhœa, diphtheria, etc., etc., as the disease may chance to be that the Board is in quest of a father for. The whole of this involves a contradiction difficult for the skeptical mind to comprehend.

The long-received theory of etiology of intermittent was marsh miasma—vegetable decay, under the influence of wet and heat, evolving a *specific* cause. But many acute observers have taken note of the fact that intermittent has now and again made its appearance, sometimes with great malignity, where there was no marsh or vegetable decay out of which it could possibly be evolved. They have taken note of another fact, to-wit: that the regions of country are abundant where no indigenous cases of malarial have been known for two or three generations, and where, without any change in the physical features of the places, rather of a sudden intermittent and remittent have appeared and prevailed extensively. The residence of the writer is one of these places, and a typical example of a great many others. An open country town, on the banks of the Connecticut river; the village mostly on high ground, without swamps or marshes, and not behind any section of the state for healthfulness of situation; two mill-ponds in the south part of the town in the same condition for the past 75 or 100 years; the same meadows in one corner, and the like water flowing by from the mountains of New Hampshire and Vermont that has flowed since the world was; finished three generations ago; and without any change in its physical features or in its surroundings. Coming here in the spring of 1854, I had in the summer intermittent severely, with relapses. The event was as notable as could have been a cure of leprosy; the like had never been known by the oldest inhabitant. I supposed, and still suppose, that the case was an imported one, as I had lived the previous summer on a part of New York Island, where fever and ague was quite common; doubtless it had been contracted there, and remained latent the intervening nine months. Three or four other *imported* cases were seen in the next eighteen years, and in 1857 one case unmistakably indigenous, and the only native case until the summer of 1872, when there were several cases. From then on it has continued to the extent that in the nine years more than one half of the residents of the town have had intermittent fever or the allied diseases, many of them many times, some for several continuous seasons, and with great severity. And now, with this experience, I challenge the most acute investigation to show me the cause of the disease in this place, or to point to any source or condition of things out of which miasma can emanate that has not existed for the last hundred years; and I make bold to say that in ten minutes I can explode any theory that can be advanced to account for its prevalence, and convict it to be as hollow as the winds. Sewers, we have none; swamps, we have none; mill-ponds, two, in the same condition as for three generations; meadows, as for 150 years; river, as from eternal, and flowing from a region where malaria is not known, and never was known; new soil upturned, none; cleanly in our habits as we ever were; our wells as well protected; cellars and pig styes the same, and the stock of rotten garbage not increased. There is nothing about the place that can generate the cause of fever and ague that has not existed for the century past, nor about the towns surrounding it. And what is true here is true of scores of places in the western half of Connecticut, where, for the past seven to nine years intermittent has been epidemic. Now mark: there are other towns in which intermittent has prevailed, where there has been some physical cause; a brook has been diverted from its old course, a cellar has been dug, a new mill-pond has been gathered or an old one has run away, a new sewer has been constructed or an old one has got plugged, the contents of a cesspool have penetrated the soil in contact with a well, a turnip has rotted beneath the eaves, an unthrifty housewife has emptied her slop bucket around the back door, the gutter has become filled with the leaves of autumn, etc., and each and all of these in turn is *charged* with the crime of malarial poisoning, but not one of them has been *convicted* to the satisfaction of the discriminating intelligence which essays to penetrate to the ultimate cause. When a batch of intermittent is encountered around a pond newly constructed, or around the site of an old one recently emptied, the conclusion seems legitimate, and is quite irresistible, that the pond is the cause of the trouble; but when, at the same period of time, another batch of intermittent is encountered where there is no pond, nor ever has been one, nor any other something capable of generating that hypothecal potency which we call miasm, (and plenty of such are seen) the curious enquirer is stimulated to demand stronger proof than the mere evidence of propinquity, before he yields entire consent to the justice of his first conclusion. Caustic interrogation exposes the fallacy of many deductions based solely upon contiguity. Skepticism demands something more. When I cross a sluggish stream which seems to present the conditions popularly supposed to be requisite for the development of ague, and learn that the people living along it are that way afflicted, I am not surprised; but when I further learn that the banks of that stream have been peopled for two hundred years and more, and that for the last eighty there has been no change in it in the way of dams or the like, and that from the first, or certainly for three generations past, intermittent was a disease unknown until 1872, and that the trouble made its appearance in other towns around at about the same period, constrained I am to ask—If this stream, or pond, is the mother of malaria, why has it been unfruitful until now? I know just this place, and I know a hundred others the like, and being unable to answer the query in the case, I am looking for that philosopher whose sagacity is equal to the problem involved.

From personal observation and experience, and without attempting to fortify the position by quotations from men of close penetration, I presume to put it down as true, —that neither marshes, swamps, ponds, emptied reservoirs, rivers, upturned earth, vegetable decay, nor any other *known* special physical condition is *necessary* to the production of intermittent fever, or rather to the cause of it; that it exists many times and in many places independently and without the concurrence of these, they only being accessories to the development where they are located; that when the poison seems, or does proceed out of them, it is because some other unrecognized factor has previously entered in; that of themselves they are incapable of *instituting* that poison, heat and moisture being so formidable, though they become suitable dwelling places for it; that they do not *originate* the cause; and that they are barren of the original fruit until the essential potency has entered from without. Out of swamps, from around ponds, in the neighborhood of sewers, where to-day, on moderate exposure, you can get any number of intermittents, ten years ago you could not coax a single case, though you boarded and bedded there for weeks in seasons either wet or dry; and yet in all outward aspects they remain the same. And you have the same disease without the contiguity of the alleged causes.

This brings us to the place where we may make the enquiry—Will you get rid of these so-called malarial diseases by draining the marsh, by emptying the new ponds or re-filling the old one, by cleansing the sewer, and by carting off the garbage? Presumably, nine times out of ten this would receive an affirmative answer. But the question is not so easily settled. It is indeed time, it has been time a great many times, that where cases of malarial disease have occurred in the neighborhood of some marsh, and the marsh has been drained, the disease has no longer declared itself; but it is equally true,—it has been true a great many more times,—that where the same class of troubles has prevailed in the vicinity of some other marsh and the marsh has continued to remain in the same condition, malarial disorders have finally passed away, and have not returned again for generations. Exactly the same thing is true in reference to some foul sewer; there has been disease near it—the sewer is purged, and the disease no longer afflicts the people near by; but in another case where the disease has existed along-side of the foul sewers, and the sewer has been left unpurged, the disease also subsides, and quite as suddenly and completely as in the first case. It is in the experience of thousands of physicians that cases of typhoid fever, or of diphtheria, arise suddenly, attack one or more members of a family, and are not followed by other cases. In one such instance the removal of a cesspool is triumphantly advertised as as the undoubted reason why a family does not continue to be afflicted with other cases of the disease; but in a score of other instances the cesspool will remain undisturbed, while the disease vanishes just the same.

It is not my purpose to attempt the vain task of showing that there is no relation whatever between filth, in the broad sense, and the diseases that are found in contiguity to it. I shall not undertake to show, for instance, that there is never any relation between intermittent and some mysterious agency supposed to haunt low-lying valleys and reeking swamps. But I do wish to point out that the intermittent is not born *of* them, that it exists independent of, and without their agency, and that the *ultimate cause* is to be looked for beyond. I wish also to protest against that unsatisfactory conclusion that *every* case of paludal disease is the resultant of some contiguous fault in the shape of marsh or pond, hole or gutter, or congregation of filth, animal or vegetable; that its subsidence is always the legitimate outcome of the correction of that fault; and that this class of disease is to be punished by what is called proper sanitation.

Too much is assumed and too little is proven; and it is in the province of health boards to prove more and assume less; to bring to bear an analysis that shall discriminate between presumptions and proofs; to present logic that is not illogical, and theory that cannot be so easily punctured, as is this one of malarial emanations as the cause of so-called malarial troubles. Exacting science asks for something more definite as to the etiology of this group of diseases than has heretofore been presented. The ground is not covered by the old theory, nor by any new ones. There is in them all somewhere an error; and the abandonment of error is the first step towards the acquisition of truth. R. W. Griswold.

